# Effects of fat distribution on lung function in young adults

**DOI:** 10.1186/s40101-019-0198-x

**Published:** 2019-06-24

**Authors:** Liqian Huang, Ziliang Ye, Jingjing Lu, Cunqing Kong, Qingqing Zhu, Binbin Huang, Zerong Wang, Lin Xu, Qiongying Deng, Jiangu Gong, Peng Liu

**Affiliations:** 1 Department of Anatomy, Guangxi Medical University, No. 22 Shuangyong Road, Nanning, 530021 Guangxi China; 2 Guangxi Medical University, Nanning, 530021 Guangxi China

**Keywords:** Visceral adipose tissue, Subcutaneous adipose tissue, Vital capacity index, Visceral fat area, College students

## Abstract

**Aims:**

To study the associations between fat distribution and lung functions in healthy subjects of young adults and to explore potential gender difference in these correlations.

**Methods:**

A total of 2101 adult participants were recruited. Height, weight, and vital capacity index (VCI) were measured and recorded according to the national physical fitness test standard. Body compositions, including body mass index (BMI), body fat percentage (BFP), waist-to-hip ratio (WHR), fat-free mass (FFM), trunk muscle mass (TMM), fat mass (FM), visceral fat area (VFA), visceral adipose tissue (VAT), and subcutaneous adipose tissue (SAT), were conducted using body composition analyzer. Data were analyzed by SPSS 20.0 software.

**Results:**

We found that male participants showed significantly higher BMI, WHR, FFM, TMM, VFA, and VCI, but lower FM, BFP, and SAT in comparison with women. However, there was no significant difference in VAT between the male and female. Lung functions represented by VCI were negatively correlated with FM, VAT, SAT, and VFA for both men and women (*r* < 0; *P* < 0.05). Among these negative correlations, VCI was more inversely correlated with VFA for men but with SAT for women, respectively. After dividing the whole populations by BMI, BFP, and WHR, further correlation analysis showed VCI was still more negatively correlated with VFA for all male subgroups (*r* < 0; *P* < 0.05). On the contrary, VCI was more negatively correlated with SAT in BMI-underweight, BMI-normal, BFP-low fat, BFP-normal fat, WHR-normal, and WHR-obese subgroups (*r* < 0; *P* < 0.05), while VFA and VAT was more inversely correlated with VCI in BMI- and BFP-overweight+obese subgroups (*r* < 0; *P* < 0.05).

**Conclusions:**

Fat accumulation is highly associated with the vital capacity index in young adults. In general, VCI was more negatively correlated with VFA for men but with SAT for women, respectively, in comparison with other tested indices.

## Introduction

Abdominal fat distribution has been associated with increased risk of different diseases, including cardiovascular disease, type 2 diabetes, and inflammatory bowel disease [1, 2]. Increased abdominal fat distribution has been correlated with higher levels of triglyceride, total cholesterol, and low-density lipoprotein cholesterol, which contribute to subsequent high blood pressure and cardiovascular risk [3]. In a recent study, hip- and waist-specific polygenic scores representing the levels of abdominal fat have been associated with higher blood pressure and higher risk of diabetes (waist-specific score: odds ratio (OR), 1.57; hip-specific score: OR, 2.54) and coronary disease (waist-specific score: OR, 1.60; hip-specific score: OR, 1.76) [4].

Breathing is an essential function for survival, and changes in pulmonary function can affect the quality of life and performance of daily activities. Previous studies have suggested that obesity is associated with impaired respiratory functions, including reduction in total lung capacity and forced vital capacity [5–7]. Obesity is also commonly found in association with chronic airway disease, like COPD and asthma [8]. Body mass index (BMI, calculated as weight in kilograms divided by height in meters squared) that is commonly used to define overweight or obesity has been negatively associated with lung functions evaluated by vital capacity, forced expiratory volume in 1 s (FEV1), and forced vital capacity (FVC) [9, 10]. In addition, increased waist circumference (WC), waist-to-hip ratio (WHR), and body fat percentage (BFP) that are used to measure body fat distribution have been linked to impaired lung functions, which is suggestive of the essential roles of fat accumulation in lung functions [9–14].

The deposition of both subcutaneous adipose tissue (SAT) and visceral adipose tissue (VAT) determines the accumulation of abdominal fat. VAT and SAT are involved in metabolic activities and the production of pro-inflammatory adipocytokines [15]. In comparison with SAT, VAT is believed to be more important in the metabolic derangement, insulin resistance [16], dyslipidemia [17], and inflammation [18]. Since SAT and VAT differ in composition and function, it is relevant to establish the contribution of each to the association between abdominal obesity and lung function. Visceral fat, but not the SAT, WC, and BMI, has been inversely associated with FEV1 and FVC of men aged 50–70 years with the metabolic syndrome [19]. In addition, visceral adiposity is associated with the decrease in lung function in female asthma patients with a mean age of 55.39 years [20], whereas the observed associations between visceral adiposity and lung functions are limited to subjects with either metabolic syndrome or asthma, and it is unknown whether these associations are consistent in healthy subjects. Also, those correlations may be age dependent. In the current study, we recruited 2101 college students and take their advantages of being homogeneous to investigate the associations between fat distribution and lung functions. The BMI, BFP, WHR, fat-free mass (FFM), trunk muscle mass (TMM), fat mass (FM), visceral fat area (VFA), VAT, SAT, and vital capacity index (VCI, standard for lung function) were measured. We analyzed the association between lung function and VAT, SAT, and VFA as well as BMI, BFP, and WHR in young adults. The gender differences among these correlations were analyzed as well.

## Materials and methods

### Subject

**Fig. 1:**
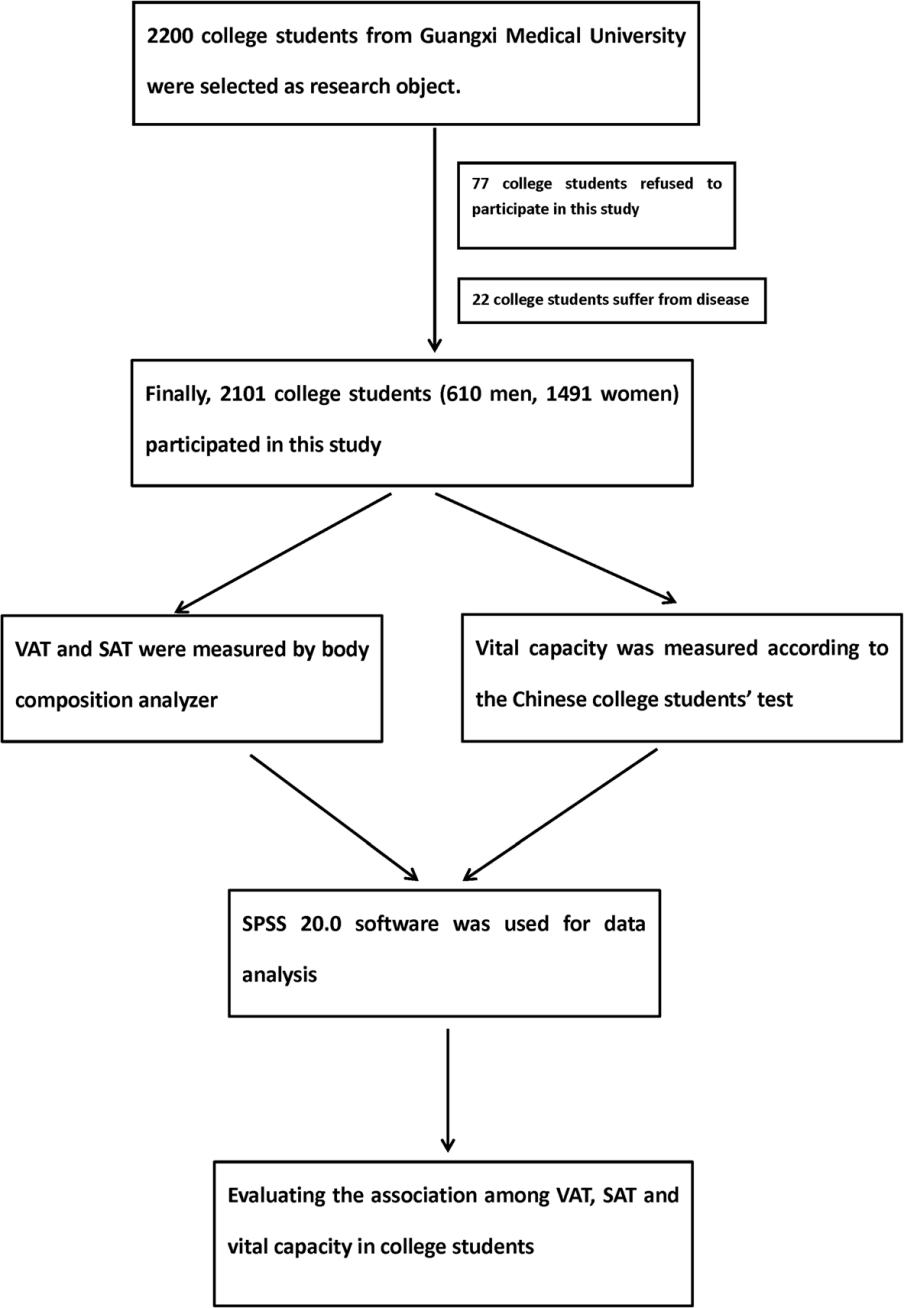
The flow chart of selecting the object of study

A total of 2101 college students over 18 years of age were recruited from Guangxi Medical University after taking the approval of the Human Research Ethics Committee. Informed consent was taken from each participant. Participants with severe liver, respiratory, and cardiovascular diseases with previous medications of glucocorticoids and anti-tuberculosis drugs and with history of trauma and surgery in the past year were excluded. According to the inclusion criteria and exclusion criteria, 99 college students out of 2200 students were excluded from our study (77 college students refused to participate in this study and 22 college students suffer from disease). The flow chart is shown in Fig. 1.

### Body composition

Anthropometric measurements were performed for individual participant wearing light clothing without shoes. Height was measured to the nearest millimeter and weight to the nearest 0.1 kg. Body composition indices were measured using bioelectrical impedance analysis (BIA) method by the body composition analyzer MC-180 (TANITA, Japan) including the BMI (expressed kg/m2), FM, BFP, WHR, FFM, TMM, VFA, VAT, and SAT. Body compositions were measured by trained professional. In addition, we divided participants into different subgroups according to the built-in evaluation criteria of Ogilvy physical fitness management system and TANITA MC-180, as shown below [21]:

- BMI-based criteria: underweight (BMI < 18.5), normal weight (18.5 ≤ BMI < 24), overweight (24 ≤ BMI < 28), and obese (BMI ≥ 28).
- WHR-based criteria: for women, normal weight (WHR < 0.8) and obese (WHR ≥ 0.81); for men, normal weight (WHR < 0.9) and obese (WHR ≥ 0.91).
- BFP-based criteria: for women, low fat (≤ 20%), normal fat (21–34%), high fat (35–39%), and obese (≥ 40%); for men, low fat (≤ 10%), normal fat (11–21%), high fat (22–26%), and obese (≥ 27%).

### Vital capacity test

The vital capacity was measured using a spirometry meter (model number: WQS-8888, Wanqing Electronics, Shanghai, China) following the guidance of the national physical health test standard. In brief, prior to performing spirometry, the equipment was calibrated and each participant’s identification was checked. Each individual was in a standing position with feet flat on floor and legs uncrossed. Tidal (normal) breaths were taken first, followed by a full inspiration along with head slightly backward. Each subject then exhaled steadily into the mouthpiece for as long as possible until there is no air left. Maximum value was recorded after three acceptable maneuver times. Detailed test methods referred to the interpretation of student physical health standard (trial scheme) published by People’s Education Press, People’s Republic of China. Since vital capacity is affected by body shape such as height and weight, we employed the vital capacity index with the consideration of the body mass, to represent the lung ventilation function [22]: the vital capacity index = vital capacity/weight [23].

### Statistical analysis

Results were presented as mean ± SD. Statistical analysis was performed using the SPSS 20.0 software. Statistical significance was set at *P* < 0.05. Statistical significance was assessed using analysis of variance (ANOVA). The confounding factors were adjusted by the covariance. Person’s correlation analysis was used to explore the relationship between body composition and vital capacity index. Controlling FFM and TMM, partial correlation analysis was used to explore the relationship between adipose tissue and vital capacity index.

## Results

The survey was completed by 2101 participants. The descriptive characteristics of body composition parameters and lung function test parameters between men and women are shown in Table 1. Male participants showed significantly higher BMI, WHR, FFM, TMM, VFA, and VCI, but lower FM, BFP, and SAT in comparison with women. However, there was no significant difference in VAT between the male and female.

**Table 1:** Characteristics of the participants

Number	Total (2101)	Men (610)	Women (1491)	*P* value
Age (years)	19.93 ± 1.468	20.02 ± 1.494	19.89 ± 1.456	0.051
Height (cm)	160.99 ± 7.305	169.01 ± 5.881	157.72 ± 4.899	0.000
Weight (kg)	51.37 ± 8.286	58.79 ± 8.866	48.33 ± 5.725	0.000
FFM	40.84 ± 7.475	50.87 ± 5.412	36.74 ± 2.957	0.000
TMM	19.56 ± 3.560	24.14 ± 2.704	17.69 ± 1.673	0.000
BMI	19.74 ± 2.312	20.54 ± 2.664	19.41 ± 2.063	0.000
FM (kg)	10.55 ± 4.319	7.95 ± 4.660	11.61 ± 3.676	0.000
BFP (%)	20.46 ± 7.039	12.87 ± 5.539	23.56 ± 4.905	0.000
WHR	0.80 ± 0.048	0.86 ± 0.377	0.78 ± 0.248	0.000
VFA (cm^2^)	18.69 ± 17.602	31.27 ± 25.563	13.54 ± 8.843	0.000
VAT (kg)	0.83 ± 0.618	0.87 ± 0.858	0.82 ± 0.486	0.167
SAT (kg)	9.70 ± 3.798	7.07 ± 3.827	10.78 ± 3.215	0.000
VCI (mL/kg)	55.89 ± 11.498	62.82 ± 11.614	53.06 ± 10.175	0.000

*BMI* body mass index, *FFM* fat-free mass, *TMM* trunk muscle mass, *FM* fat mass, *BFP* body fat percentage, *WHR* waist-to-hip ratio, *VFA* visceral fat area, *VAT* visceral adipose tissue, *SAT* subcutaneous adipose tissue, *VC* vital capacity, *VCI* vital capacity index

**Fig. 2:**
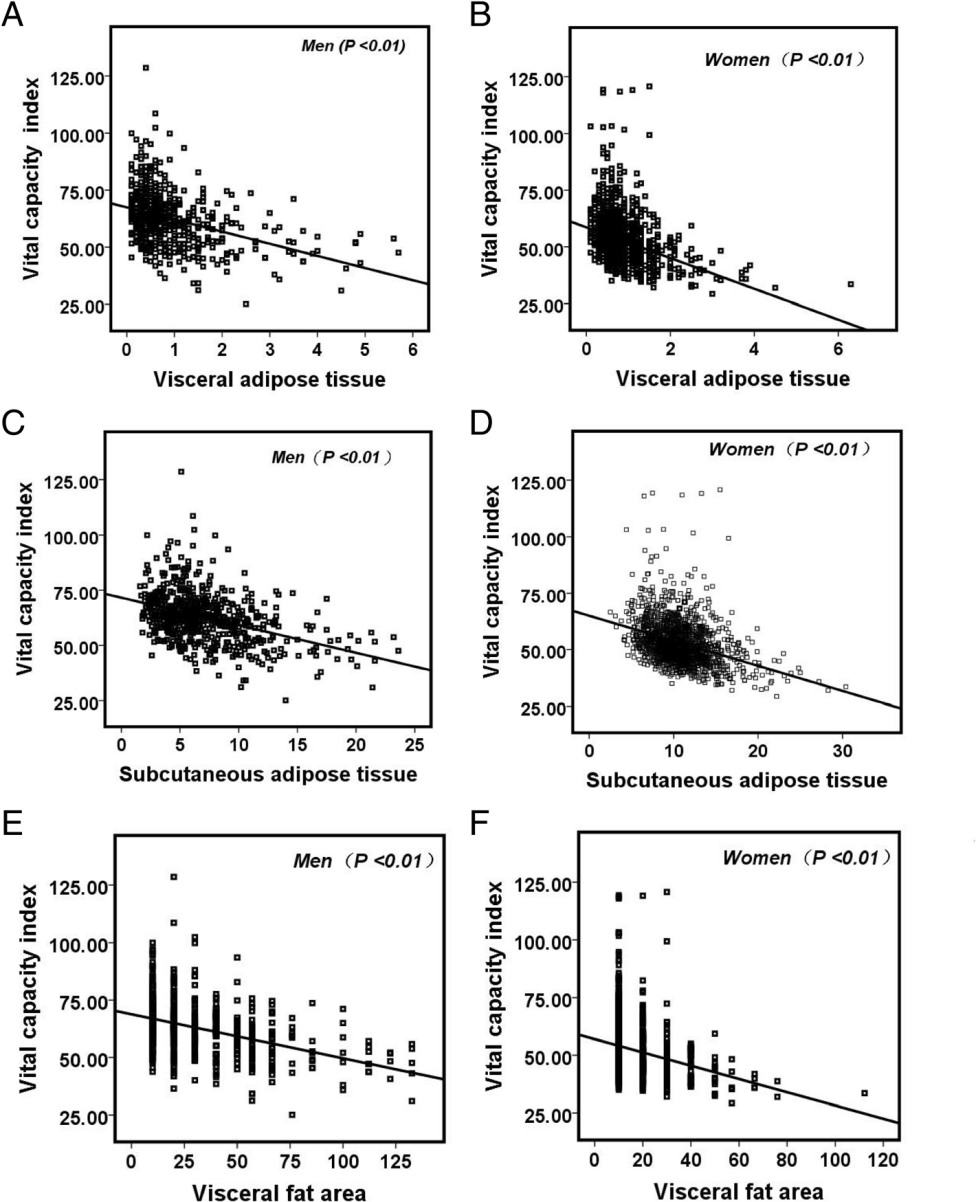
Scatter plot of the correlation between visceral adipose tissue (**a**, **b**), visceral fat area (**e**, **f**), subcutaneous adipose tissue (**c**, **d**), and vital capacity index (**a**-**f**). They all showed a negative correlation with the amount of vital capacity index, *P* < 0.01 indicated that negative correlation was significant

Next, we explored the association between VCI and body composition indices. In Table 2, Pearson’s correlation analysis showed, for men, negative correlations between VCI and FM, FFM, TMM, VAT, SAT, BMI, BFP, and WHR, respectively (*r* < 0, *P* < 0.01). Women showed similar result as men, and FM, FFM, TMM, VAT, SAT, BMI, BFP, and WHR were negatively correlated with VCI (*r* < 0, *P* < 0.01). As shown in Fig. 2, the scatter plot analysis showed negative correlations between VAF, VAT, and SAT, and VCI in both men and women, respectively.

**Table 2:** Pearson’s correlation analysis between body composition indices and vital capacity index

	Men			Women	
	*r*	*P* value		*r*	*P* value
FFM	− 0.332	< 0.01		− 0.213	< 0.01
TMM	− 0.308	< 0.01		− 0.162	< 0.01
FM	− 0.409	< 0.01		− 0.350	< 0.01
VFA	− 0.423	< 0.01		− 0.250	< 0.01
VAT	− 0.394	< 0.01		− 0.323	< 0.01
SAT	− 0.411	< 0.01		− 0.351	< 0.01
BMI	− 0.450	< 0.01		− 0.352	< 0.01
BFP	− 0.382	< 0.01		− 0.331	< 0.01
WHR	− 0.373	< 0.01		− 0.337	< 0.01

*FFM* fat-free mass, *TMM* trunk muscle mass, *FM* fat mass, *VFA* visceral fat area, *VAT* visceral adipose tissue, *SAT* subcutaneous adipose tissue, *BMI* body mass index, *BFP* body fat percentage, *WHR* waist-to-hip ratio

Since muscle mass and FFM have significant association with pulmonary function, we further performed partial correlation analysis after controlling FFM and TMM [24, 25]. As shown in Table 3, the partial correlation analysis showed that VCI was negatively correlated with FM, VFA, VAT, and SAT, respectively, after controlling for FFM and TMM for both men and women (*r* < 0, *P* < 0.01). The strength of these negative correlations was VFA > SAT > FM > VAT (*r* < 0, *P* < 0.01) for men, suggesting VFA was more negatively correlated with male VCI. While for women, the strength of these negative correlations was SAT > FM > VAT > VFA (*r* < 0, *P* < 0.01), which suggested SAT was more inversely correlated with female VCI. Taken together, these results suggested that VCI and fat distribution are negatively correlated for both men and women. Compared to other indices, VCI was more negatively correlated with VFA for men but SAT for women, respectively.

**Table 3:** Partial correlation analysis between adipose tissue and vital capacity index

Controlling variables		Men		Women	
		*r*	*P* value	*r*	*P* value
FFM and TMM	FM	− 0.290	< 0.01	− 0.281	< 0.01
	VFA	− 0.294	< 0.01	− 0.182	< 0.01
	VAT	− 0.274	< 0.01	− 0.256	< 0.01
	SAT	− 0.292	< 0.01	− 0.282	< 0.01

*FM* fat mass, *VFA* visceral fat area, *VAT* visceral adipose tissue, *SAT* subcutaneous adipose tissue

To illustrate these observations, we further divided the men (Table 4) and women (Table 5) into BMI-base, BFP-based, and WHR-based subgroups, separately. Because of the limited case numbers for both BMI-obese group and BFP-obese group, we combined overweight group and obese group together for analysis. For men, negative correlations between VCI and FM, VAF, VAT, and SAT were significant in BMI-normal, BFP-normal fat, and WHR-normal subgroups (*r* < 0, *P* < 0.01), but not for other subgroups. Based on the correlation strength (Table 4), VCI was more negatively correlated to VFA in each of these subgroups, in comparison with other indices of body fat distribution.

**Table 4:** Partial correlation analysis between adipose tissue and vital capacity index in BMI-, BFP-, and WHR- based male subgroups

		BMI					BFP					WHR	
		Underweight	Normal	Overweight	Obese	Overweight + obese	Low fat	Normal fat	High fat	Obese	Overweight + obese	Normal	Obese
Controlling variables	*n*=	136	408	52	14	66	260	306	30	14	44	527	83
FFM and TMM	FM	− 0.136	− 0.231**	− 0.068	0.004	− 0.198	− 0.053	− 0.187**	− 0.094	− 0.329	− 0.108	− 0.203**	− 0.175
	VAF	< − 0.001	− 0.237**	− 0.082	− 0.174	− 0.233	− 0.083	− 0.193**	− 0.012	− 0.548	− 0.120	− 0.200**	− 0.192
	VAT	− 0.146	− 0.235**	− 0.070	0.019	− 0.199	− 0.048	− 0.179**	− 0.123	− 0.291	− 0.106	− 0.198**	− 0.156
	SAT	− 0.136	− 0.231**	− 0.067	− 0.011	− 0.199	− 0.052	− 0.191**	− 0.078	− 0.355	− 0.110	− 0.204**	− 0.180

*FM* fat mass, *VFA* visceral fat area, *VAT* visceral adipose tissue, *SAT* subcutaneous adipose tissue, *BMI* body mass index, *BFP* body fat percentage, *WHR* waist-to-hip ratio.

**P < 0.01, with statistical significance

**Table 5:** Partial correlation analysis between adipose tissue and vital capacity index in BMI-, BFP-, and WHR-based female subgroup

		BMI					BFP					WHR	
		Underweight	Normal	Overweight	Obese	Overweight + obese	Low fat	Normal fat	High fat	Obese	Overweight + obese	Normal	Obese
Controlling variables	*n*=	472	977	35	7	42	424	1041	21	5	26	527	83
FFM and TMM	FM	− 0.195**	− 0.165**	− 0.397*	− 0.816	− 0.421**	− 0.163**	− 0.147**	− 0.164	− 0.607	− 0.214	− 0.251**	− 0.275**
	VAF	− 0.028	− 0.108**	− 0.405*	− 0.873	− 0.428**	< − 0.001	− 0.108**	− 0.006	− 0.668	− 0.241	− 0.094**	− 0.257**
	VAT	− 0.170*	− 0.160**	− 0.407*	− 0.841	− 0.413**	− 0.137**	− 0.145**	0.006	− 0.574	− 0.245	− 0.233**	− 0.280**
	SAT	− 0.196**	− 0.165**	− 0.391*	− 0.810	− 0.421**	− 0.164**	− 0.145**	− 0.172	− 0.616	− 0.204	− 0.251**	− 0.272**

*FM* fat mass, *VFA* visceral fat area, *VAT* visceral adipose tissue, *SAT* subcutaneous adipose tissue, *BMI* body mass index, *BFP* body fat percentage, *WHR* waist-to-hip ratio

*P < 0.05; **P <  0.01, all with statistical significance

For women, as shown in Table 5, negative correlations between VCI and FM, VAF, VAT, and SAT were significant for the BMI-normal, BMI-overweight+obese, BFP-normal fat, WHR-normal, and WHR-obese subgroups (*r* < 0, *P* < 0.05). For BMI-underweight group and BFP-low fat group, VCI had a significant negative correlation with FM, VAT, and SAT (*r* < 0, *P* < 0.05), but not VAF. The correlation coefficient (Table 5) indicated a relative strong negative relation between VCI and SAT in BMI-underweight, BMI-normal, BFP-low fat, BFP-normal fat, and WHR-normal. On the contrary, VAF and VAT were more inversely correlated with VCI in BMI-overweight, BMI -overweight+obese, and WHR-obese subgroups.

## Discussion

In the current study, we recruited 2101 college students to study the effects of abdominal fat distribution on lung function in young adults. We found that VCI was negatively correlated with FM, VAT, SAT, and VFA through partial correlation analysis after controlling muscle mass. Among these negative correlations, VCI was more inversely correlated with VFA for men but with SAT for women, respectively. Further correlation analysis for BMI-, BFP-, and WHR-subgroups suggested VCI was more negatively correlated to VFA in each of these male subgroups, in comparison with other indices of body fat distribution. However, for women, strong negative correlations between VCI and SAT were observed only in BMI-underweight, BMI- normal, BFP-low fat, and BFP-normal fat subgroups. On the contrary, for BMI- and BFP-overweight+obese subgroups, VCI was more negatively correlated with VAF or VAT. We concluded that fat distribution is highly associated with lung function in young adults, and in general, VCI was more negatively correlated with VFA for men but with SAT for women, respectively, in comparison with other tested indices.

In addition to cardiovascular disease, type 2 diabetes, and inflammatory bowel disease, abdominal fat distribution has been associated with impaired lung functions. Similar to other diseases [26, 27], different abdominal fat compartments and distributions may be differently associated with lung functions. However, only a few studies have assessed the independent associations of VAT and SAT with lung functions. Visceral fat, but not the SAT, WC, and BMI, has been inversely associated with impaired lung function of men aged 50–70 years with the metabolic syndrome [19]. In addition, visceral adiposity is associated with the decrease in lung function in female asthma patients with a mean age of 55.39 years [20]. These observations were obtained from the participants with the ages over 50 years and subjected to limitations of health conditions (metabolic syndrome or asthma). It is unclear whether these associations are universal and applicable to healthy people. To address this question, we recruited college students in this study and take their advantages to investigate the association between fat accumulation and lung function. College student subjects are believed to be developmentally mature both physically and psychologically. In addition, they tend to be homogeneous on dimensions such as age, education, dwelling, and food source, as well as exercise behavior. Therefore, college student subjects might enhance research validity and minimize the possibility of undue influence because of their apparent homogeneity, especially for the research purpose targeting on young adults.

Dual energy X-ray absorptiometry (DXA) and bioelectrical impedance analysis are two common methods to determine body composition. DXA method is featured with high accuracy and has been recognized as a gold standard technique to measure human body composition [28, 29]. DXA can be used to determine either whole or regional body composition. However, this standard method is hardly feasible in routine clinical practice, especially in our current study with over 2000 participants. In addition, the radiation exposure from DXA may cause potential participants’ psychological concerns and subsequently unwillingness to be involved in this project, though the method is safe. Therefore, in this study, we chose BIA method instead which has been considered inexpensive and rapid [30]. Some studies have shown good concordance between the two methods while others have not [31–39]. These conflicting results may probably be due to the differences in used equations, population size, age, ethnicity, gender, and body weights in the sample studied. A recent study that directly compares the measurement of FM and FFM by DXA and BIA methods in a large cohort of patients suggested that BIA and DXA methods are interchangeable at a population level and FM obtained by BIA and DXA were strongly correlated [31]. In particular, BMI between 16 and 18.5, body composition values measured by DXA and BIA were very closed. However, BIA method tends to overestimate or underestimate FM and FFM values beyond the above BMI range.

Sex differences in fat distribution are well documented. Women are generally characterized by having more SAT, whereas men are more prone to high amounts of VAT [40–42], which is consistent with our finding although the VAT difference between men and women was not statistically significant. Of note, we also showed men had higher VFA than women. Gender is considered as an essential factor regulating the body composition, in particular the obesity, mostly upon genetic and epigenetic regulation [43]. Other biological differences between male and female also contribute to the different composition [44], including hormone expression and lifestyle. For example, estrogen could increase the decomposition of SAT but decrease VAT [45].

Jianhui et al. have found that the effects of different fat distribution on thoracic activity varied; central obesity tended to reduce diaphragm and chest activity significantly, while peripheral obesity had relatively slight effects on respiratory movement [46]. Since the fat distribution varies in men and women, it is reasonable that gender difference in fat distribution contributes differently to lung functions for men and women, in addition to the gender difference in lung morphology (lung size, airway diameter, and diffusion surface) and hormones [47–50]. In this study, VCI was negatively correlated with FM, VFA, VAT, and SAT, respectively, for both men and women. However, women’s VCI was more negatively correlated with SAT, while male VCI was more inversely correlated with VFA, suggesting a gender difference of effects of fat distribution on lung functions, confirming our hypothesis. Such observation is probably due to the fact that compared with subcutaneous fat, the accumulation is more visceral for men [51], and the increase of visceral fat is faster, resulting in excessive accumulation of fat in the internal organs of the abdominal cavity and affecting lung ventilation function for male [52]. Therefore, visceral fat had a predominant impact on lung function than subcutaneous fat for young men. For women, a previous study by Park et al. showed that VAT rather than SAT is more important for pulmonary function in female with an average age of 53.4 years [53]. The difference may come from different employed indicators for lung function, VCI in our study vs FVC and FEV1 in the previous report. Another possibility is the age difference. Considering the fact that postmenopausal women tend to distribute fat in their viscera [54], it is plausible that the age difference contributed to the contradiction with the previous report.

To better understand these observed negative correlations, we performed further analysis by dividing participants into different subgroups based on the BMI, BFP, and WHR criteria. For men, VCI was more negatively correlated to VFA regardless of subgroups, in comparison with other indices of body fat distribution, suggesting VCI-VFA negative correlation is universal for the whole population. This is probably due to the fact that visceral fat deposition is predominant in young men regardless of obesity classification as discussed above [51]. On the contrary, female VCI was more negatively correlated with SAT in BMI-underweight, BMI-normal, BFP-low fat, BFP-normal fat, and WHR-normal subgroups, which is consistent with the finding as a whole female population. VFA and VAT were more inversely correlated with VCI in BMI-overweight, BMI-overweight+obese, and WHR-obese subgroups. Taken together, for women, the fat distribution indices of different subgroups have different effects on the lung functions. It is plausible that female’s fat is mainly deposited under the skin but slowly in viscera before the body turning into overweighted or obese, which contributes to a negative correlation between SAT and VCI. However, during the development of obesity, fat tends to accumulate in visceral region since the amount of subcutaneous fat has plateaued. A previous study showed that obese women with high visceral fat have worse lung function compared to those with high subcutaneous fat under the same fat content [55]. Further studies or animal models are needed to illustrate the potential mechanism.

In addition to VAT, VFA, and SAT, we also showed that the BMI, FM, BFP, and WHR were negatively correlated with the vital capacity index. The increase of BMI, body fat rate, and WHR has been related to the development of obesity, whose increase is indicative of excessive fat accumulation in the abdominal cavity and on the chest wall. These changes will impair pulmonary function by affecting vital capacity and breathing regulation, as well as increasing the work of breathing, reducing lung volumes, rendering respiratory muscles dysfunctional, and impairing gas exchange [56–58]. In the present study, we also noticed gender differences in BMI, BFP, and WHR and the vital capacity index. Women’s FM and BFP were higher than men, while men’s BMI, WHR, and vital capacity index were higher than women.

The present study had several limitations. Firstly, lung function was only evaluated by vital capacity index due to a large sample size. FVC, FEV1, and FEV1/FVC are considered for later studies to establish the relationship between fat accumulation with other lung function indices. Secondly, because this study is cross-sectional, our study lacks the analysis about the causality underlying the relationship between fat accumulation and impaired lung function. Future experiment is expected to explain the effects of SAT and VAT on lung functions.

## Conclusion

Taken together, we conclude that lung function is highly associated with abdominal fat distribution in young adults. Female and male VCIs are more negatively correlated with SAT and VFA separately.

## Data Availability

The datasets used during the current study are available from the corresponding authors on reasonable request.
